# Comprehensive analysis of Japanese nationwide cohort data of particle beam therapy for pulmonary, liver and lymph node oligometastases: particle beam therapy versus high-precision X-ray radiotherapy

**DOI:** 10.1093/jrr/rrad004

**Published:** 2023-04-13

**Authors:** Norihiro Aibe, Hiroyuki Ogino, Masaru Wakatsuki, Kei Fujikawa, Satoshi Teramukai, Nobuyoshi Fukumitsu, Shintaro Shiba, Naoyoshi Yamamoto, Akihiro Nomoto, Takashi Ono, Masanosuke Oguri, Hisashi Yamaguchi, Haruko Numajiri, Kei Shibuya, Shohei Okazaki, Yuhei Miyasaka, Noriyuki Okonogi, Kazutoshi Murata, Hitoshi Tatebe, Atsushi Motegi, Tomoaki Okimoto, Takayuki Yoshino, Masaki Mandai, Norio Katoh, Hiroshi Tsuji, Hideyuki Sakurai

**Affiliations:** Department of Radiology, Kyoto Prefectural University of Medicine, Kyoto 602-8566, Japan; Department of Radiation Oncology, Nagoya Proton Therapy Center, Nagoya City University West Medical Center, Nagoya 462-8508, Japan; QST Hospital, National Institutes for Quantum Science and Technology, Chiba 263-8555, Japan; Department of Biostatistics, Kyoto Prefectural University of Medicine, Kyoto 602-8566, Japan; Department of Biostatistics, Kyoto Prefectural University of Medicine, Kyoto 602-8566, Japan; Department of Radiation Oncology, Kobe Proton Center, Hyogo 650-0047, Japan; Department of Radiation Oncology, Gunma University Graduate School of Medicine, Gunma 371-8511, Japan; QST Hospital, National Institutes for Quantum Science and Technology, Chiba 263-8555, Japan; QST Hospital, National Institutes for Quantum Science and Technology, Chiba 263-8555, Japan; Department of Radiation Oncology, Yamagata University, Faculty of Medicine, Yamagata 990-9585, Japan; Department of Radiation Oncology, Nagoya Proton Therapy Center, Nagoya City University West Medical Center, Nagoya 462-8508, Japan; Department of Radiology, Southern Tohoku Proton Therapy Center, Koriyama City, Fukushima 963-8052, Japan; Department of Radiation Oncology, University of Tsukuba, Ibaraki 305-8550, Japan; Department of Radiation Oncology, Gunma University Graduate School of Medicine, Gunma 371-8511, Japan; Department of Radiation Oncology, Gunma University Graduate School of Medicine, Gunma 371-8511, Japan; Department of Radiation Oncology, Gunma University Graduate School of Medicine, Gunma 371-8511, Japan; QST Hospital, National Institutes for Quantum Science and Technology, Chiba 263-8555, Japan; QST Hospital, National Institutes for Quantum Science and Technology, Chiba 263-8555, Japan; Proton Therapy Center, Fukui Prefectural Hospital, Fukui 910-0846, Japan; Department of Radiation Oncology, National Cancer Center Hospital East, Chiba 277-8577, Japan; Department of Radiology, Hyogo Ion Beam Medical Center, Hyogo, Japan; Department of Gastrointestinal Oncology, National Cancer Center Hospital East, Chiba, Japan; Department of Gynecology and Obstetrics, Kyoto University Graduate School of Medicine, Kyoto 606-8501, Japan; Department of Radiation Oncology, Hokkaido University Faculty of Medicine, Hokkaido 060-0808, Japan; QST Hospital, National Institutes for Quantum Science and Technology, Chiba 263-8555, Japan; Department of Radiation Oncology, University of Tsukuba, Ibaraki 305-8550, Japan

**Keywords:** particle beam therapy (PT), proton beam therapy (PBT), carbon ion radiotherapy, metastasis-directed therapy, oligometastasis

## Abstract

Japanese national oncological experts convened to evaluate the efficacy and safety of particle beam therapy (PT) for pulmonary, liver and lymph node oligometastases (P-OM, L-OM and LN-OM, respectively) and to conduct a statistically comparative analysis of the local control (LC) rate and overall survival (OS) rate of PT versus those of X-ray stereotactic body radiotherapy (X-SBRT) and X-ray intensity-modulated radiotherapy (X-IMRT). They conducted [1] an analysis of the efficacy and safety of metastasis-directed therapy with PT for P-OM, L-OM and LN-OM using a Japanese nationwide multi-institutional cohort study data set; [2] a systematic review of X-ray high-precision radiotherapy (i.e. X-SBRT/X-IMRT) and PT for P-OM, L-OM and LN-OM; and [3] a statistical comparison between LC and OS of the cohort data set in PT and that of the extracted historical data set in X-SBRT/X-IMRT from the preceding systematic review. Safety was evaluated as the incidence of grade ≥ 3 adverse events, while statistical comparisons of LC and OS were conducted by estimating the incidence rate ratios (IRR) for local progression and mortality, respectively. This study demonstrated that PT provided durable LC (3-year LC rate: 72.8–83.2%) with acceptable OS (3-year OS rate: 38.5–68.1%) and risk of severe toxicity incidence of 0.8–3.5% in radical metastasis-directed therapy for P-OM, L-OM and LN-OM. Compared to LC with X-SBRT or X-IMRT, LC with PT was potentially superior for P-OM; superior for L-OM; and equivalent for LN-OM. In particular, this study demonstrated that PT may be a new treatment option for L-OM tumors measuring > 5 cm.

## INTRODUCTION

Increasing evidence supports the concept of oligometastases [1], which was first proposed by Hellman and Weichselbaum [2], as a cancer state that is an intermediate metastatic state between localized disease and systemically metastasized disease for which local metastasis-directed therapies have the potential of prolonging survival. X-ray high-precision radiotherapy (i.e. stereotactic body radiotherapy [X-SBRT] and/or intensity-modulated radiotherapy [X-IMRT]), a local metastasis-directed therapy, is widely used, based on promising clinical evidence [3–5]. The American Society for Radiation Oncology (ASTRO) model policies indicate X-SBRT is a suitable local metastasis-directed therapy [6], and the Japanese national health insurance system has covered X-SBRT for treating oligometastatic disease since April 2020. However, limited evidence exists regarding particle beam therapy (PT), including proton beam therapy (PBT) and carbon-ion radiotherapy (C-ion RT), for treating oligometastatic disease. PT for oligometastatic disease is not indicated as a suitable treatment, except for PT of metastatic tumors of the spine [7], and it is not covered by the national health insurance system in Japan. However, PT has excellent therapeutic results, owing to the physical characteristics of the Bragg peaks [8], and it has great potential as an optimal metastasis-directed treatment for oligometastatic disease. Therefore, informative reports on the outcomes of PT for oligometastatic disease are needed to accumulate evidence on the usefulness of PT for oligometastatic disease.

In Japan, a nationwide multi-institutional cohort study on PT started in all Japanese centers with PBT and/or C-ion RT in May 2016. Several years have passed since the nationwide cohort study began, and the Japanese Society for Radiation Oncology (JASTRO) decided to conduct a comprehensive analysis of PT for pulmonary oligometastasis (P-OM), liver oligometastasis (L-OM) and lymph node oligometastasis (LN-OM). The aim of this study was (i) to analyze the efficacy and toxicity of PT by using the Japanese multi-institutional cohort study data set; (ii) to conduct a systematic review on X-SBRT/X-IMRT and PT in radical metastasis-directed therapy for P-OM, L-OM and LN-OM; and (iii) to conduct a statistical comparison between the outcomes of the cohort data in PT and the outcomes of the historical data in X-SBRT/X-IMRT, which were extracted from a previous systematic review. These studies were conducted and managed by the Oligometastatic Cancer Working Group in the Particle Beam Therapy Committee and Subcommittee at JASTRO, which involves radiation oncologists from JASTRO, oncologists of the Japan Society of Clinical Oncology, and biostatisticians. In this study, we present the results of the aforementioned investigations on P-OM, L-OM and LN-OM.

## MATERIALS AND METHODS

The following three analyses were conducted individually for the analysis of P-OM, L-OM and LN-OM. This study was approved by the appropriate institutional ethics committees.

### Analysis of the Japanese multi-institutional cohort study data set of PT

Among patients registered in a Japanese multi-institutional cohort study, the data of patients who received PBT or C-ion RT between May 2016 and June 2018 were reviewed to evaluate the local control (LC) rate, OS rate and grade ≥ 3 treatment-related late toxicity. The main eligibility criteria were as follows: (i) histopathological or clinical diagnosis of oligometastatic disease; (ii) number of metastatic tumors ≤3 for P-OM and L-OM, and localized metastatic region for LN-OM; (iii) absence of recurrence at the primary disease site after primary curative treatment; (iv) absence or control of other cancers and clinically detectable recurrent or metastatic diseases other than the metastatic regions; and (v) curative-intent PBT or C-ion RT to all metastatic regions. The details of the criteria are summarized in Table 1.

**Table 1:** The inclusion criteria for the analyses on the Japanese multi-institutional cohort study data set of PT or C-ion RT

**Pulmonary oligometastatis**
Histopathological or clinical diagnosis of pulmonary metastasis Number of metastatic lung tumors ≤ 3 Absence of recurrence in primary disease site after primary curative treatment Absence or control of the other cancers and clinically detectable recurrent or metastatic diseases other than the metastatic lung tumors Delivery of PBT or C-ion RT to all metastatic lung tumors with curative intent between May 2016 and June 2018 Total irradiation dose was 64 Gy (RBE)/8fr., 66 Gy (RBE)/10fr., 70-80 Gy (RBE)/22-35fr. in PBT and 50 Gy (RBE)/1fr., 60 Gy (RBE)/ 4fr., 69.6-72 Gy (RBE)/12-16fr. in C-ion RT, depending on the tumor location.
**Liver oligometastasis**
Histopathological or clinical diagnosis of liver metastasis Number of metastatic liver tumors ≤ 3 Absence of recurrence in primary disease site after primary curative treatment Absence or control of the other cancers and clinically detectable recurrent or metastatic diseases other than the metastatic liver tumors Delivery of PBT or C-ion RT to all metastatic liver tumors with curative intent between May 2016 and June 2018 Total irradiation dose was 64 Gy (RBE)/8fr., 66 Gy (RBE)/10fr., 72.6-76 Gy (RBE)/20-38fr. in PBT and 58 Gy (RBE)/1fr., 60 Gy (RBE)/4fr., 64-76 Gy (RBE)/8-20fr. in C-ion RT, depending on the tumor location.
**Lymph node oligometastasis**
Histopathological or clinical diagnosis of lymph node metastasis Localized metastatic lesion of the lymph nodes Absence of recurrence in primary disease site after primary curative treatment Absence or control of the other cancers and clinically detectable recurrent or metastatic diseases other than the metastatic lymph node diseases Delivery of PBT or C-ion RT to all metastatic lymph node lesions with curative intent between May 2016 and June 2018 BED_10_ of the delivery dose ≥ 60 Gy (RBE). Patients who did not receive PBT two or more times for the treatment of two or more lymph node legions, because the cohort data set of PBT was difficult to calculate the LC rate among such patients.

Abbreviations: PBT, proton beam therapy; C-ion RT, carbon-ion radiotherapy; RBE, relative biological effectiveness; fr, fractions; BED_10,_ biological effective dose using the linear-quadratic model with α/β = 10 Gy.

LC was defined as the time from the initiation of PT to the progression of treated lesions. OS was defined as the time from PT initiation to death from any cause. Treatment-related late toxicity of grade ≥ 3, defined as complications appearing more than 3 months after the end of PT, was evaluated by using the Common Terminology Criteria for Adverse Events (version 4.0) [9]. LC and OS probabilities were estimated using the Kaplan–Meier method, and survival curves were compared using the log-rank test. To evaluate the impact of a prescribed dose on LC, the survival curves of PT with a prescribed dose < the median value or ≥ the median value were compared, using the log-rank test. Furthermore, among the L-OM series, the efficacy and toxicity of PT for tumors measuring > 5 cm and tumors measuring ≤5 cm were evaluated with statistical comparisons. All statistical analyses were conducted using JMP Pro16 (SAS Institute Inc., Cary, NC, USA) or SAS 9.4 (SAS Institute Inc.). All reported *P*-values were two sided, and *P* < 0.05 was considered statistically significant.

### Systematic review of X-SRBT/X-IMRT and PT

Systematic literature reviews on P-OM, L-OM and LN-OM were conducted individually and in accordance with the Preferred Reporting Items for Systematic Reviews and Meta-Analyses (PRISMA) guidelines [10]. The PubMed electronic database was searched for clinical scientific reports on X-SBRT/X-IMRT or PT for P-OM, L-OM and LN-OM published in English between January 2000 and September 2020. The search terms, selection criteria, data collection and other details of these systematic reviews are provided in Supplementary Data 1. In each review, two radiation oncologists independently reviewed the retrieved articles and selected potentially relevant articles, based on their titles and abstracts. In addition to this screening, the two experts conducted a manual search, as needed, to select other relevant articles for the full-text review. Finally, full-text reviews were conducted to identify studies that met the selection criteria.

### Comparison between the cohort data for PT and the extracted historical data for X-SBRT/X-IMRT, based on the systematic review

To compare the endpoint of interest (i.e. local progression or mortality) between the cohort data of PT (i.e. PBT and C-ion RT) and historical data extracted from the systematic review of X-ray therapy (i.e. X-SBRT/X-IMRT), the incidence rate ratio (IRR) of the endpoint of interest was evaluated. ${N}_i$, ${M}_i$ and ${E}_i$ denote the number of subjects (in the case of local progression, the number of target sites), the median follow-up time and the number of events, respectively, for *i* = 1 and *i =* 2 (*i* = 1 represents the X-ray therapy group and *i* = 2 represents the particle therapy group). ${N}_1$, ${M}_1$ and ${E}_1$ were calculated by summing the reports of all X-ray therapy articles in the analysis, assuming that they were randomly sampled from the same population. When data on the number of events were missing, they were imputed from the survival probability, under the assumption of an exponential distribution. The IRR was estimated as follows: $IRR=\frac{\frac{E_2}{N_2\times{M}_2}}{\frac{E_1}{N_1\times{M}_1}}$, in which IRR < 1 indicates that the incidence rate of the endpoint of interest is lower in the particle therapy group than in the X-ray therapy group. The 95% confidence interval (CI) of the IRR was estimated and tested with IRR = 1 as the null hypothesis.

Analytical methods and results have varied among the selected studies on X-ray therapy (i.e. X-SBRT/X-IMRT) for P-OM, L-OM and LN-OM. Some reports focused on one specific primary cancer (specific primary cancer article), while other studies reported the outcomes of summary data on various primary cancers without revealing the individual outcomes of each primary cancer. In the current study, a statistical comparison was principally conducted between all data of PT (i.e. PBT and C-ion RT) and all data of X-ray therapy (i.e. X-SBRT/X-IMRT).

Furthermore, when possible, certain statistical comparisons were conducted between the same specific primary cancer data sets of X-ray therapy and PT. The comparisons included only data focusing on the specific primary cancer to minimize the impact of primary cancer on the values of interest (i.e. local progression rate or mortality).

## RESULTS

### Pulmonary oligometastasis

#### Analysis of the Japanese multi-institutional cohort study data set of PT

A total of 132 patients (representing 156 tumors) were eligible, based on the inclusion criteria. Among them, 85 patients received PBT at 107 sites, while 47 patients received C-ion RT at 49 sites. The median patient age was 69 years (range, 25–88 years). The patients’ and treatment characteristics are shown in Table 2 and Supplementary Data 2. The median follow-up period was 27.9 months (range, 1.6–54.7 months). The major primary disease sites were the colorectum (*n* = 48, 36.4%) and lungs (*n* = 35, 26.5%). The median biological effective dose obtained, using the linear-quadratic model with α/β = 10 Gy (biologic effective dose, BED_10_), was 115.2 Gy (relative biological effectiveness [RBE]; range, 84–300 Gy [RBE]).

**Table 2:** Characteristics of patients and treatments

Characteristics	P-OM 132 patients (156 tumors)			L-OM 200 patients (266 tumors)			LN-OM 282 patients (287 regions)	
Age, years Median (range)	69 (25–88)			68 (24–90)			66 (36–96)	
Sex, n (%) Male Female	74 (56.1) 58 (43.9)			125 (62.5) 75 (37.5)			148 (52.5) 134 (47.5)	
Performance status^*^, n (%) 0–1 2 unknown	130 (98.5) 2 (1.5) 0 (0.0)			191 (95.5) 8 (4.0) 1 (0.5)			272 (96.5) 7 (2.5) 3 (1.0)	
Follow-up time, months Median (range)	27.9 (1.6–54.7)			20.2 (1.0–55.9)			24.2 (2.5–56.4)	
Primary disease, n (%)	Colorectal cancer Lung cancer Esophageal cancer Kidney cancer Uterine cancer Breast cancer Bone & soft tissue tumor Head & Neck cancer The others	48 (36.4) 35 (26.5) 8 (6.1) 8 (6.1) 7 (5.3) 7 (5.3) 4 (3.0) 4 (3.0) 11 (8.3)		Colorectal cancer Biliary tract cancer Pancreatic cancer Stomach cancer Breast cancer Esophageal cancer Lung cancer Small intestine cancer The others	102 (51.0) 28 (14.0) 17 (8.5) 15 (7.5) 9 (4.5) 6 (3.0) 5 (2.5) 4 (2.0) 14 (7.0)		Colorectal cancer Uterine cancer Lung cancer Esophageal cancer Pancreatic cancer Head & Neck cancer Biliary tract cancer Ovary cancer Stomach cancer Hepatocellular cancer Breast cancer Prostate cancer Kidney cancer Small intestine cancer Bladder cancer The others	43 (15.2) 38 (13.5) 37 (13.1) 28 (9.9) 23 (8.2) 23 (8.2) 14 (5.0) 13 (4.6) 11 (3.9) 10 (3.5) 9 (3.2) 7 (2.5) 4 (1.4) 4 (1.4) 2 (0.7) 16 (5.7)
Tumor size, mm Median (range)	15 (5-130)			29.5 (7-170) ^**^			NA	
Treatment modality, n (%) PBT C-ion RT	85 (64.4) 47 (35.6)			151 (75.5) 49 (24.5)			205 (72.7) 77 (27.3)	
Total delivery dose, Gy (RBE) Median (range)	64 (50–80)			66 (58–76)			60 (48–74)	
Dose per fraction, Gy (RBE) Median (range)	8 (2–50)			6.6 (2–58)			2.5 (2–6.6)	
Number of treatment fractions, n Median (range)	8 (1–35)			10 (1–38)			26 (10–37)	
BED_10_, Gy (RBE) Median (range)	115.2 (84–300)			109.6 (72–394.4)			79.2 (60–109.6)	

Abbreviations: P-OM, pulmonary oligometastasis; L-OM, liver oligometastasis; LN-OM, lymph node oligometastasis; RBE, relative biological effectiveness; PBT, proton beam therapy; C-ion RT, carbon-ion radiotherapy; BED_10,_ biological effective dose using the linear-quadratic model with α/β = 10 Gy; NA, not available

^*^According to the Eastern Cooperative Oncology Group.

^**^The values were described based on the available registry data of 250 tumors.

**Fig. 1:**
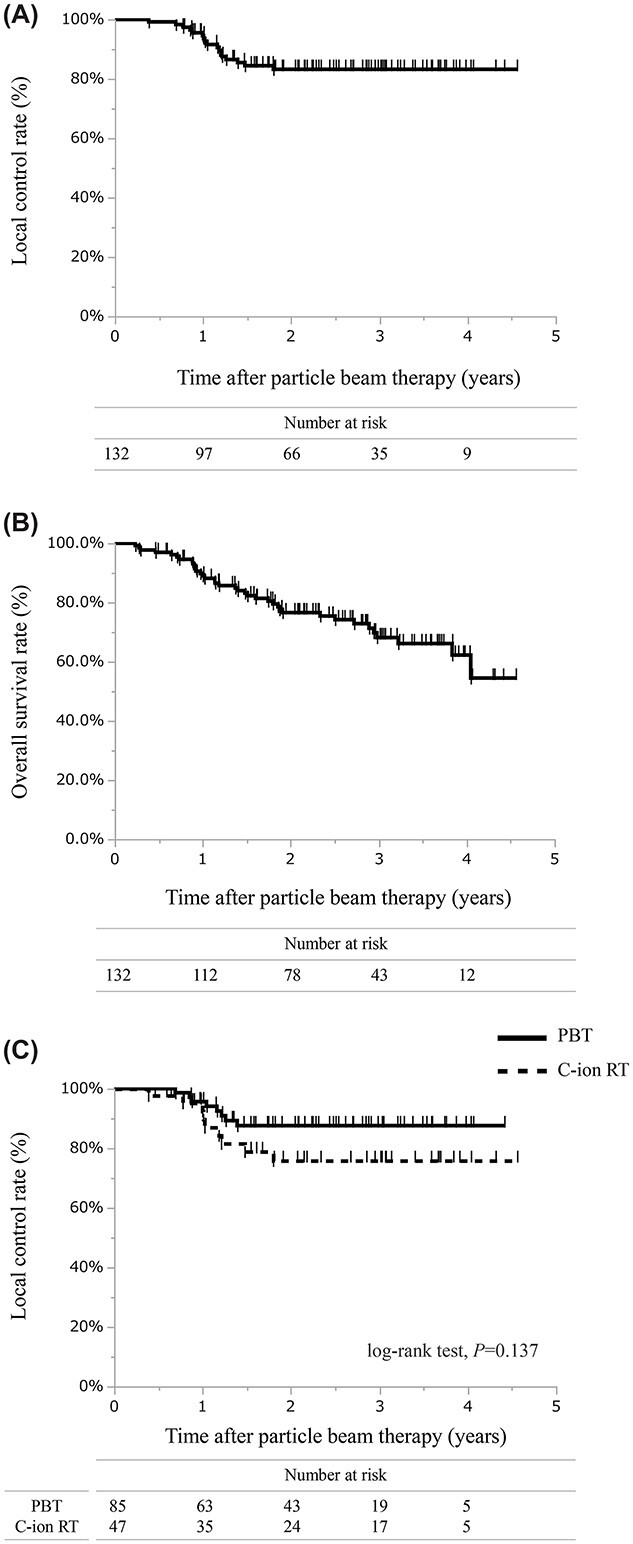
Survival curves of PT for P-OM. (**A**) LC rate of all data. (**B**) OS rate of all data. (**C**) LC rates of PBT and C-ion RT.

At the last follow-up, 17 (12.9%) patients had local progression, whereas 37 (28%) patients died of any cause. In the statistical analysis of P-OM, the number of patients with local progression, but not the number of relapse lesions, was used to calculate the LC because of the difficulty in discriminating which lesion relapsed when the patient had received treatment for multiple lesions that were close to each other. In all patients, the 1-, 2- and 3-year LC rates were 94.5% (95% CI, 90.3–98.8), 83.2% (95% CI, 75.9–90.5) and 83.2% (95% CI, 75.9–90.5), respectively (Fig. 1A). The 1-, 2- and 3-year OS rates were 89% (95% CI, 83.5–94.4), 76.6% (95% CI, 68.9–84.2) and 68.1% (95% CI, 58.7–77.5), respectively (Fig. 1B). The median survival was not reached until this analysis. For LC, no significant difference existed between PBT and C-ion RT (log-rank test, *P =* 0.14) (Fig. 1C) and between PT series with the prescribed dose < the median value or ≥ the median value (log-rank test, *P* = 0.18).

Only 1 (0.8%) treatment-related grade 3 late toxicity occurred among the 132 patients treated with PT: the patient had radiation pneumonitis. No grade ≥ 4 treatment-related late toxicity occurred.

#### Systematic review of X-SBRT and PT

The PRISMA flow diagram of the systematic review of X-SBRT and PT for P-OM is shown in Supplementary Data 1 (Fig. S1). In total, five relevant studies on X-SBRT [11–15] and three relevant studies on PT [16–18] were identified (Table 3). Different studies had different inclusion criteria, characteristics of patients and primary cancers, and treatment protocols. Among the five reports on X-SBRT, one study [11] was prospectively conducted, whereas one study [15] was a meta-analysis focusing on the P-OM of colorectal cancer. All reports on PT were retrospective studies with small sample sizes (< 120). The 2- and 3-year LC rates, 2- and 3-year OS rates and incidence of grade ≥ 3 toxicities were extracted from relevant reports and are listed in Table 3. The LC and OS rates were obtained from Kaplan–Meier curves, if necessary.

**Table 3:** Relevant articles of the systematic review on X-SBRT and PT for P-OM

Authors (Reported year)	Study design	Treatment methods	Number of patients	Primary cancer	LC (%)	OS (%)	G ≥ 3 AEs (%)
Ricco A [11] (2017)	M/P	X-SBRT	447	Various primary sites	64 (2 Y) 59 (3 Y)	57 (2 Y) 33 (3 Y)	NA
Niibe Y [12] (2020)	M/R	X-SBRT	1378	Various primary sites	NA (2 Y) 81 (3 Y)	70 (2 Y) 60 (3 Y)	2.2
De Rose F [13] (2016)	S/R	X-SBRT	60	Lung cancer	89 (2 Y) 45 (3 Y)	75 (2 Y) 64 (3 Y)	3.3
Yamamoto T [14] (2020)	M/R	X-SBRT	330	Colorectal cancer	67 (2 Y) 63 (3 Y)	80 (2 Y) 65 (3 Y)	2.0
Choi HS [15] (2020)	MA	X-SBRT	495	Colorectal cancer	72 (2 Y) 56 (3 Y)	70 (2 Y) 58 (3 Y)	NA
Aibe N [16] (2021)	M/R	PBT	118	Various primary sites	86 (2 Y) 78 (3 Y)	68 (2 Y) 60 (3 Y)	0.8
Yamamoto N [17] (2013)	S/R	C-ion RT	91	Various primary sites	92 (2 Y) 88 (3 Y)	71 (2 Y) 62 (3 Y)	0
Takahashi W [18] (2014)	S/R	C-ion RT	44	Colorectal cancer	85 (2 Y) 85 (3 Y)	65 (2 Y) 50 (3 Y)	0

Abbreviations: PT, particle beam therapy; P-OM, pulmonary oligometastasis; X-SBRT, X-ray stereotactic body radiotherapy; PBT, proton beam therapy; C-ion RT, carbon-ion radiotherapy; S, single-institutional investigation; M, multi-institutional investigation; R, retrospective design; P, prospective design; MA, meta-analysis; LC, local control rate; OS, overall survival rate; G ≥ 3 AEs, adverse effects of grade ≥ 3; Y, years; NA, not available.

#### Comparison between the cohort data for PT and the extracted historical data for X-SBRT, based on the systematic review

Among the five relevant studies selected by a systematic review on X-SBRT for P-OM, the data from four studies [11–14] were used to make statistical comparisons with the Japanese cohort data of PT. However, the data of one study [15] was not used because it was a meta-analysis of X-SBRT and did not describe the number of events that were necessary for statistical comparison. The statistical comparisons were conducted in the following combinations: (i) between all data sets of X-SBRT and PT and [2] between the same specific primary cancer data sets (e.g. colorectal cancer and lung cancer) of X-SBRT and PT. The results of statistical comparison between all data sets are listed in Table 4. The results between the same specific primary cancer data sets are shown in Supplementary Data 3. The statistical comparison between all data sets revealed a statistically significant difference in LC (IRR, 0.56; 95% CI, 0.34–0.91) and in OS (IRR, 0.66; 95% CI, 0.47–0.92). However, the statistical comparisons between the same specific primary cancer data sets (e.g. colorectal cancer and lung cancer) revealed no significant difference in LC and in OS.

**Table 4:** Results of comparison between the all data sets of the cohort data of PT and those of the extracted historical data of X-SBRT/X-IMRT, based on the systematic review

	Treatment modality (Number of patients / target sites)	Local progression		Mortality		Incidence of G ≥ 3 AEs (%)
		IRR (95% CI)	*P* value	IRR (95% CI)	*P* value	
**P-OM**	PT (132/ 156)	0.56 (0.34–0.91)	0.020	0.66 (0.47–0.92)	0.014	0.8
	X-SBRT (2215/ 2335)					1.5–3.3
**L-OM**	PT (200/ 266)	0.52 (0.37–0.72)	< 0.001	0.97 (0.78–1.20)	0.778	3.5
	X-SBRT (809/ 1071)					0–9.8
**LN-OM**	PT (282/ 287)	0.82 (0.61–1.11)	0.200	1.17 (0.96–1.43)	0.122	3.2
	X-SBRT/X-IMRT (1070/ 1267)					0–21.1

Abbreviations: PT, particle beam therapy; P-OM; pulmonary oligometastasis; L-OM, liver oligometastasis; LN-OM, lymph node oligometastasis; PBT, proton beam therapy; C-ion RT, carbon-ion radiotherapy; X-SBRT, X-ray stereotactic body radiotherapy; G ≥ 3 AEs, adverse effects of grade ≥ 3; IRR, incidence rate ratio; CI, confidence interval.

### Liver oligometastasis

#### Analysis of the Japanese multi-institutional cohort study data set of PT

Two hundred patients were eligible (representing 266 tumors). Among them, 151 patients received PBT at 208 sites, while 49 patients received C-ion RT at 58 sites. The median patient age was 68 years (range, 24–90 years). The patients’ and treatment characteristics are shown in Table 2 and Supplementary Data 2. The median follow-up period was 20.2 months (range, 1.0–55.9 months). The major primary disease sites were the colorectum (*n* = 102, 51%), biliary tract (*n* = 28, 14%), pancreas (*n* = 17, 8.5%) and stomach (n = 17, 7.5%). The median BED_10_ was 109.6 Gy (RBE) [range, 72–394.4 Gy (RBE)].

**Fig. 2:**
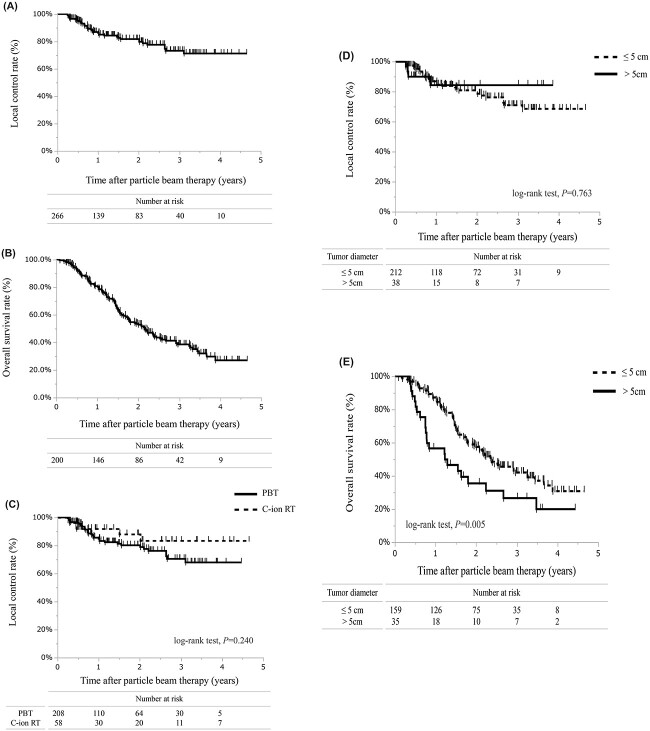
Survival curves of PT for L-OM. (**A**) LC rate of all data. (**B**) OS rate of all data. (**C**) LC rates of PBT and C-ion RT. (**D**) LC rates of PT for a target size of ≤ 5 cm and > 5 cm. (**E**) OS of PT for a target size of ≤ 5 cm and > 5 cm.

At the last follow-up, local progression occurred in 41 lesions in 266 treated sites (15.4%, 41/266), while 111/200 (55.5%) patients died of any cause. Among all irradiated liver lesions, the 1-, 2- and 3-year LC rates were 86.2% (95% CI, 81.4–91.2), 81.8% (95% CI, 75.9–87.6) and 73.2% (95% CI, 65.2–81.2), respectively (Fig. 2A). The 1-, 2- and 3-year OS rates were 80.8% (95% CI, 75.1–86.4), 52.9% (95% CI, 45.5–60.2) and 38.5% (95% CI, 30.-946.2), respectively (Fig. 2B). The median survival time was 25.9 months. For LC, no significant difference existed between PBT and C-ion RT (log-rank test, *P* = 0.24) (Fig. 2C) or between PT series with a prescribed dose < the median value or ≥ the median value (log-rank test, *P* = 0.26).

Seven (3.5%) of 200 patients had grade 3 treatment-related late toxicities. No grade ≥ 4 treatment-related late toxicity occurred. Grade 3 toxicities included radiation dermatitis in 3 patients, anemia in 1 patient, ascites in 1 patient, gamma-glutamyl transferase increase in 1 patient and unknown toxicity in 1 patient.

This cohort data set included available data on the maximum tumor diameter of 250 tumors in 194 patients. Among them, 159 patients with 212 lesions had tumors measuring ≤5 cm (i.e. small-size group) and 35 patients with 38 lesions had tumors measuring > 5 cm (i.e. large-size group). The 1-, 2- and 3-year LC rates of the small-size group were 86.1% (95% CI, 80.7–91.4), 80.8% (95% CI, 74.3–87.3) and 70.9% (95% CI, 61.9–80.0), respectively. The 1-, 2- and 3-year LC rates of the large-size group all had the same value at 84.5% (95% CI, 69.8–99.1). No statistical difference existed between the LC rates of the two groups (log-rank test, *P* = 0.76) (Fig. 2D). However, the OS rates among these groups differed significantly (log-rank test, *P* < 0.01) (Fig. 2E). The 1-, 2- and 3-year OS rates of the small-size group were 87.2% (95% CI, 81.8–92.6), 57.4% (95% CI, 49.2–65.6) and 42% (95% CI, 33.2–50.8), respectively. These rates in the large-size group were 56.5% (95% CI, 39.4–73.7), 35.5% (95% CI, 18.2–52.7) and 26.6% (95% CI, 9.8–43.3), respectively. The incidence of grade ≥ 3 adverse events in the small-size group was 3.9% and in the large-size group was 2.9%.

#### Systematic review on X-SBRT and PT

The PRISMA flow diagram of the systematic review of X-SBRT and PT for L-OM is shown in Supplementary Data 1 (Fig. S2). Overall, 11 studies [19–29] on X-SBRT and 6 studies [30–35] on PT were identified (Table 5). Different studies used different inclusion criteria, characteristics of patients and primary cancers, and treatment protocols. Among the 11 reports on X-SBRT, seven studies [19, 20, 23, 25–28] were prospectively conducted, and four studies [26–29] focused on the treatment of colorectal cancer. For PT, only one prospective study [1] existed, whereas the other studies [30, 32–35] were retrospective investigations. The LC and OS rates, median survival time and incidence of grade ≥ 3 toxicities are listed in Table 5.

**Table 5:** Relevant articles of the systematic review on X-SBRT and PT for L-OM

Authors (Reported year)	Study design	Treatment methods	Number of patients	Primary cancer	LC (%)	OS (%)	G ≥ 3 AEs (%)
Kavanagh BD [19] (2006)	S/P	X-SBRT	21	Various primary sites	93 (1.5 Y)	NA	4.8
Iwata H [20] (2010)	S/P	X-SBRT	12	Various primary sites	67 (1 Y)	NA	0
Scorsetti M [21] (2013)	S/R	X-SBRT	61	Various primary sites	94 (1 Y)	MST: 19 M 83.5/65(1Y/1.5Y)	0
Yamashita H [22] (2014)	M/R	X-SBRT	51	Various primary sites	64 (2Y)	71.9 (2Y)	9.8
Ahmed KA [23] (2016)	S/P	X-SBRT	22 11	Colorectal cancer The other primary sites	79/59 (1Y/2Y) 100/100 (1Y/2Y)	100/73 (1Y/2Y) 82/73(1Y/2Y)	NA
Mahadevan A [24] (2018)	M/R	X-SBRT	427	Various primary sites	MLCT: 51 M	MST:22 M	NA
Scorsetti M [25] (2018)	S/P	X-SBRT	61	Various primary sites	94/78/78 (1Y/3Y/5Y)	MST: 27.6 85.2/31.1/18 (1Y/3Y/5Y)	1.6
Comito T [26] (2014)	S/P	X-SBRT	41	Colorectal cancer	95/90/81 (1Y/2Y/3Y)	78/61/44 (1Y/2Y/3Y)	0
Scorsetti M [27] (2015)	S/P	X-SBRT	42	Colorectal cancer	95/91/85 (1Y/2Y/3Y)	MST: 29 M 65 (2Y)	0
McPartlin A [28] (2017)	S/P	X-SBRT	51	Colorectal cancer	50/32/26 (1Y/2Y/4Y)	MST: 16 M 63/26/9 (1Y/2Y/4Y)	0
Klement RJ [29] (2019)	M/R	X-SBRT	255	Colorectal cancer	NA	MST: 27.9 M	NA
Fukumitsu N [30] (2015)	S/R	PBT	133	Various primary sites	NA	MST: 19.2 M	1.5
Makishima H [31] (2019)	S/P	C-ion RT	29	Colorectal cancer	NA	MST: 65 M 78 (3Y)	6.9
Shiba S [32] (2021)	S/R	C-ion RT	11	Colorectal cancer	61 (2Y)	100 (2Y)	0
Fukumitsu N [33] (2017)	S/R	PBT	9	Stomach cancer	71 (3Y)	78 (3Y)	0
Yamaguchi H [34] (2020)	S/R	PBT	7	Stomach cancer	85.7 (3Y)	MST: 42 M 68.6 (3Y)	0
Fukumitsu N [35] (2017)	S/R	PBT	8	Breast cancer	73 (3 Y)	86 (3 Y)	0

Abbreviations: PT, particle beam therapy; L-OM, liver oligometasitasis; X-SBRT, X-ray stereotactic body radiotherapy; X-IMRT, x-ray intensity-modulated radiotherapy; PBT, proton beam therapy; C-ion RT, carbon-ion radiotherapy; S, single-institutional investigation; M, multi-institutional investigation; R, retrospective design; P, prospective design; LC, local control rate; OS, overall survival rate; G ≥ 3 AEs, adverse effects of grade ≥ 3; MST, median survival time; MLCT, median local control time; M, months; Y, years; NA, not available.

#### Comparison between the cohort data for PT and the extracted historical data for X-SBRT, based on the systematic review

Eleven X-SBRT studies [19–29] selected by a systematic review for L-OM were all used to make statistical comparisons with the Japanese cohort data of PT. The statistical comparisons were in the following combinations: (i) between all data sets of X-SBRT and PT; and (ii) between the same specific primary cancer data sets (e.g. colorectal cancer) of X-SBRT and PT. The results of the statistical comparison between all data sets and the same specific primary cancer data sets are listed in Table 4 and Supplementary Data 3, respectively. For LC, all comparative analysis in the aforementioned combinations demonstrated that PT achieved significantly lower IRR: in the statistical comparison of all data sets, the IRR was 0.52 (95% CI, 0.37–0.72). In the same specific primary cancer data sets of colorectal cancer, the IRR was 0.33 (95% CI, 0.22–0.50). For mortality, a statistical significance existed in the comparison between the colorectal cancer data sets on X-SBRT and PT (IRR, 0.69; 95% CI, 0.49–0.97), while no statistical significance existed in the statistical comparison of all data sets.

### Lymph node oligometastasis

#### Analysis of the Japanese multi-institutional cohort study data set of PT

A total of 282 patients (representing 287 regions) were included. Among them, 205 patients received PBT in 205 regions, while 77 patients received C-ion RT in 82 regions. The median patient age was 66 years (range: 36–96 years). The patients’ and treatment characteristics are shown in Table 2 and Supplementary Data 2. The median follow-up period was 24.2 months (range: 2.5–56.4 months). The major primary disease sites were the colorectum (*n* = 43, 15.2%), uterus (*n* = 38, 13.5%), lungs (*n* = 37, 13.1%) and esophagus (*n* = 28, 9.9%). The median BED_10_ was 79.2 Gy (RBE) [range, 60–109.6 Gy (RBE)].

**Fig. 3:**
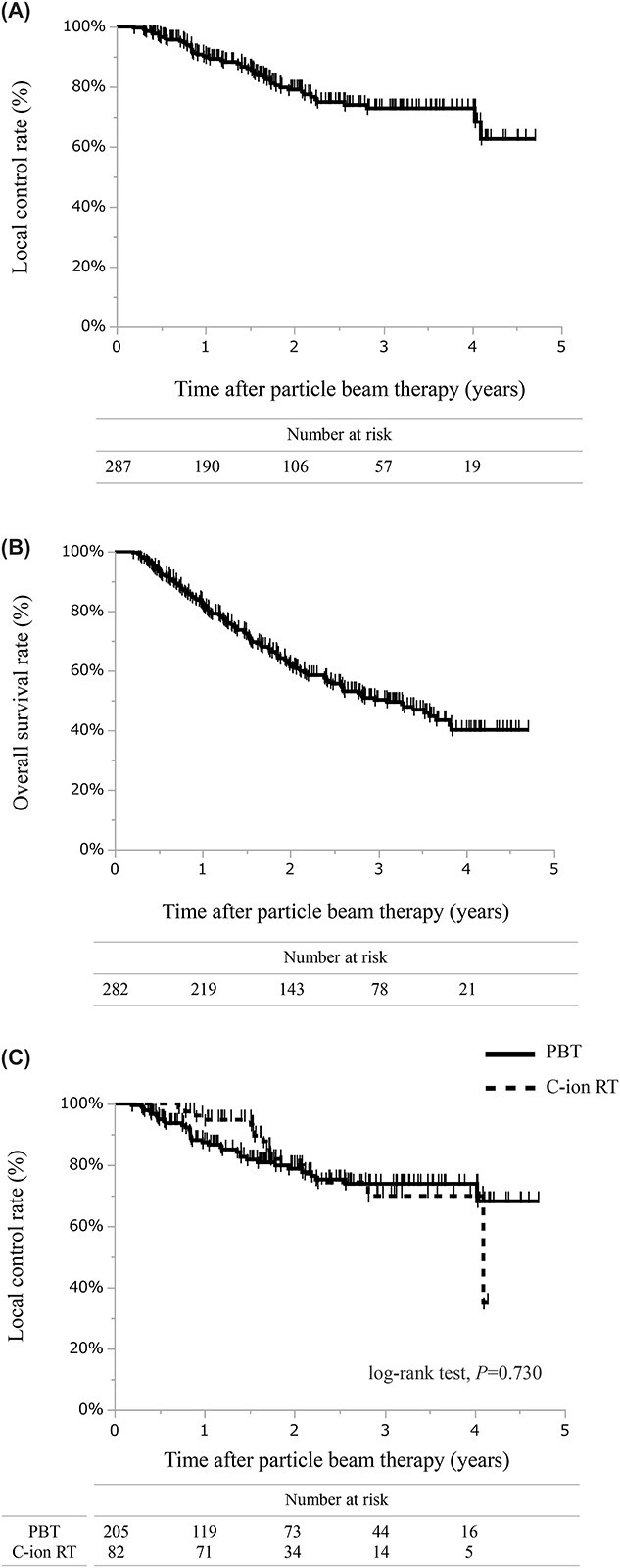
Survival curves of the PBT for LN-OM. (**A**) LC rate of all data. (**B**) OS rate of all data. (**C**) LC rates of PBT and C-ion RT.

At the last follow-up, local progression occurred in 51 regions in the treated sites among the 287 metastatic lymph node regions (17.8%, 51/287). Overall, 132/282 (46.8%) patients died of any cause. Across all irradiated lymph node sites, the 1-, 2- and 3-year LC rates were 89.8% (95% CI, 85.8–96.7), 79% (95% CI, 73.2–84.9) and 72.8% (95% CI, 65.8–79.8), respectively (Fig. 3A). The 1-, 2- and 3-year OS rates were 82.5% (95% CI, 78.1–87), 62.1% (95% CI, 56.1–68) and 50.2% (95% CI, 43.7–56.7), respectively (Fig. 3B). The median survival time was 37.2 months. For LC, no significant difference existed between PBT and C-ion RT (log-rank test, *P* = 0.73) (Fig. 3C) or between PT series with a prescribed dose < the median value or ≥ the median value (log-rank test, *P* = 0.88).

Among the 282 PT patients, nine (3.2%) patients developed grade 3 treatment-related late toxicities. No grade ≥ 4 treatment-related late toxicity occurred. Grade 3 toxicities included gastroduodenal ulcer in three patients, proctitis in one patient, pelvic abscess in one patient, urinary obstruction in one patient, tracheal fistula in one patient, vertebral fracture in one patient and peripheral neuropathy in one patient.

#### Systematic review of X-SBRT/X-IMRT and PT

The PRISMA flow diagram of the systematic review of X-SBRT/X-IMRT and PT for LN-OM is shown in Supplementary Data 1 (Fig. S3). The review identified 17 relevant studies on X-SBRT/X-IMRT [36–52] and five relevant studies on PT [53–57], respectively (Table 6). No high-level investigations were identified. Different studies had different inclusion criteria, characteristics of patients and primary cancers, and treatment protocols. Among the 17 studies on X-SBRT/X-IMRT, two studies [41, 42] were conducted prospectively, and the other studies were conducted retrospectively. However, all studies on PT were conducted retrospectively. The 2- and 3-year LC rates, 2- and 3-year OS rates and incidence of grade ≥ 3 toxicities were extracted from the relevant reports and are listed in Table 6. The LC and OS rates were extracted from published Kaplan–Meier curves, as necessary.

**Table 6:** Relevant articles of the systematic review on X-SBRT/X-IMRT and PT for LN-OM

Authors (Reported year)	Study design	Treatment methods	Number of patients	Primary cancer	LC (%)	OS (%)	G ≥ 3 AEs (%)
Aoki T [36] (2003)	S/R	X-IMRT	29	Various primary sites	58 (2 Y) 58 (3 Y)	29 (2 Y) 18 (3 Y)	0
Jereczek-Fossa BA [37] (2014)	S/R	X-SBRT	69	Various primary sites	68 (2 Y) 64 (3 Y)	59 (2 Y) 50 (3 Y)	1.4
Franzese C [38] (2016)	S/R	X-SBRT	71	Various primary sites	63 (2 Y) 63 (3 Y)	77 (2 Y) 77 (3 Y)	0
Loi M [39] (2018)	S/R	X-SBRT	91	Various primary sites	78 (2 Y) 78 (3 Y)	65 (2 Y) 52 (3 Y)	0
Ito M [40] (2020)	M/R	X-SBRT X-IMRT (3DCRT)	159	Various primary sites	60 (2 Y) NA (3 Y)	63 (2 Y) NA (3 Y)	9.8
Franzese C [41] (2020)	S/P	X-SBRT	52	Various primary sites	82 (2 Y) 82 (3 Y)	94 (2 Y) 84 (3 Y)	0
Franzese C [42] (2020)	S/P	X-SBRT	278	Various primary sites	77 (2 Y) 75 (3 Y)	74 (2 Y) 67 (3 Y)	0.4
Sato A [43] (2020)	S/R	X-IMRT	21	Various primary sites	52 (2 Y) 52 (3 Y)	89 (2 Y) 75 (3 Y)	NA
Bae SH [44] (2012)	S/R	X-SBRT	18	Colorectal cancer	NA (2 Y) NA (3 Y)	NA (2 Y) NA (3 Y)	11.1
Franzese C [45] (2017)	S/R	X-SBRT	35	Colorectal cancer	75 (2 Y) 75 (3 Y)	81 (2 Y) 81 (3 Y)	0
Ho JC [46] (2015)	S/R	X-IMRT	38	Uterine cancer	NA (2 Y) NA (3 Y)	71 (2 Y) NA (3 Y)	21.1
Park HJ [47] (2015)	M/R	X-SBRT	85	Uterine cancer	83 (2 Y) NA (3 Y)	58 (2 Y) NA (3 Y)	5.9
Choi CW [48] (2009)	S/R	X-SBRT (3DCRT)	30	Uterine cancer	76 (2 Y) 67 (3 Y)	71 (2 Y) 65 (3 Y)	3.3
Meng MB [49] (2015)	S/R	X-SBRT	33	Lung cancer	NA (2 Y) 86 (3 Y)	52 (2 Y) 41 (3 Y)	6.1
Manabe Y [50] (2018)	S/R	X-SBRT	14	Lung cancer	90 (2 Y) 81 (3 Y)	40 (2 Y) 40 (3 Y)	3.7
Ost P [51] (2016)	M/R	X-SBRT	72	Prostate cancer	NA (2 Y) 94 (3 Y)	NA (2 Y) 96 (3 Y)	0
Franzese C [52] (2017)	S/R	X-SBRT	26	Prostate cancer	75 (2 Y) 75 (3 Y)	100 (2 Y) 100 (3 Y)	0
Isozaki Y [53] (2017	S/R	C-ion RT	34	Colorectal cancer	70 (2Y) 70 (3 Y)	83 (2 Y) 63 (3 Y)	0
Shiba S [54] (2017)	S/R	C-ion RT	16	Gynecological cancer	94 (2 Y) 94 (3 Y)	74 (2 Y) 74 (3 Y)	0
Isozaki Y [55] (2018)	S/R	C-ion RT	10	Esophageal cancer	92 (2 Y) 92 (3 Y)	58 (2 Y) 58 (3 Y)	0
Okonogi N [56] (2019)	M/R	C-ion RT	323	Various primary sites	85 (2 Y) 79 (3 Y)	87 (2 Y) 63 (3 Y)	0.3
Shirai K [57] (2019)	S/R	C-ion RT	15	Lung cancer	92 (2 Y) 92 (3 Y)	75 (2 Y) 60 (3 Y)	0

Abbreviations: PT, particle beam therapy; LN-OM, lymph node oligometastasis; X-SBRT, X-ray stereotactic body radiotherapy; X-IMRT, x-ray intensity-modulated radiotherapy; PBT, proton beam therapy; C-ion RT, carbon-ion radiotherapy; S, single-institutional investigation; M, multi-institutional investigation; R, retrospective design; P, prospective design; LC, local control rate; OS, overall survival rate; G ≥ 3 AEs, adverse effects of grade ≥ 3; 3DCRT, 3-demensional conformal radiation therapy; Y, years; NA, not available.

#### Comparison between the cohort data for PT and the extracted historical data for X-SBRT/X-IMRT, based on the systematic review

Among the 17 relevant studies selected by systematic review on X-SBRT/X-IMRT for LN-OM, the data of 16 articles [36–43, 45–52] were used to make a statistical comparison with the Japanese cohort data of PT. However, the data of one study [44] was not used because it did not describe the number of events necessary for statistical comparison. The statistical comparisons were conducted using the following combinations: (i) all data sets of X-SBRT/X-IMRT and PT; and (ii) between the same specific primary cancer data sets (e.g. colorectal cancer, uterine cancer and lung cancer) of X-SBRT/X-IMRT and PT. The results of statistical comparison between all data sets and the same specific primary cancer data sets are listed in Table 4 and Supplementary Data 3, respectively. For LC, the statistical comparisons showed no significant differences between all combinations. For OS, statistical comparisons revealed no significant differences between each combination, except for the combination of the same specific lung cancer data sets.

## DISCUSSION

The aim of this study was: (i) to analyze the efficacy and toxicity of PT by using the Japanese multi-institutional cohort study data set; (ii) to conduct a systematic review on X-SBRT/X-IMRT and PT in radical metastasis-directed therapy for P-OM, L-OM and LN-OM; and (iii) to conduct a statistical comparison between the outcomes of the cohort data in PT and the outcomes of the historical data in X-SBRT/X-IMRT. Our study, which used Japanese nationwide cohort data sets, demonstrated that PT provided durable LC (3-year LC rate, 72.8–83.2%) with acceptable OS (3-year OS rate, 38.5–68.1%) and risk of severe toxicity (incidence, 0.8–3.5%) in radical metastasis-directed therapy for P-OM, L-OM and LN-OM. The analysis also showed the potential of PT as a promising treatment modality for L-OM tumors measuring > 5 cm. Furthermore, this study presented the results of statistical comparisons between the national cohort data sets of PT and the historical data sets of X-SBRT/X-IMRT. To the best of our knowledge, this study is the first using such comparisons between the data sets of PT and X-ray radiotherapy (i.e. X-SBRT/X-IMRT) for the radical metastasis-directed treatment of P-OM, L-OM and LN-OM.

### Pulmonary oligometastasis

The analysis of the cohort study data set of PT showed durable LC (3-year LC, 83.2%) and OS (3-year OS, 68.1%) with an acceptable risk of grade ≥ 3 toxicity (incidence rate, 0.8%) in metastasis-directed therapy for P-OM. This result corresponded with the results of previous reports on PT for P-OM [16–18]. The range of the prescribed dose in this cohort series was relatively wide because the prescribed doses depended on the physician’s decisions, based on the size, number and location of a target, surrounding organs at risk (e.g. the proximity of a target to organs at risk and the tolerance of organs at risk), etc. However, the statistical analysis on the impact of the prescribed dose on LC revealed no statistical significance. The reason for this finding may be that 86.4% (114/132) of lesions in the cohort series received PT with adequate delivery dose of BED_10_ > 100 Gy (RBE). Some investigations have demonstrated that the delivery dose of BED_10_ > 100 Gy was associated with better LC [11, 14]. With regard to adverse events, the PT cohort data set demonstrated that only one patient developed grade 3 pneumonitis and no patient developed grade ≥ 4 toxicity. The incidence rate was lower than that in previous data on X-SBRT (0.8% vs 1.5–3.3%) [12–14], although both rates were acceptably low.

The statistical comparison between all data of PT and X-SBRT showed significantly better LC and OS with PT. This result implied that PT is superior to X-SBRT as the treatment modality for P-OM. However, caution is needed in interpretation because the statistical subgroup analyses on the same primary cancer data sets revealed no significant difference in LC or OS. This inconsistent result may be explained by several biases. The sample size of the PT series was smaller than that of X-SBRT series. The comparison with all data sets may unintentionally have several biases. In the analysis, no adjustment was conducted in the different parameters between the PT series and X-SBRT series because of the difficulty in appropriate adjustment. However, the sample size of the same primary cancer data sets seemed to be too small to conduct a statistical comparison with sufficient accuracy. These several factors may have caused the inconsistency. Therefore, a more accurate comparison should be conducted with a larger sample size with adjustment for several elements (e.g. patient and disease characteristics, combined treatment modality and follow-up duration).

Our analysis had some limitations, although the statistical comparisons did not demonstrate that the outcome of PT for P-OM was worse than the outcome of X-SBRT. Therefore, a conclusion is that PT for P-OM demonstrated promising outcomes with the possibility that PT is superior to X-SBRT in the radical metastasis-directed treatment for P-OM.

### Liver oligometastasis

The analysis of the cohort study data set of PT showed a durable LC (3-year LC, 73.2%) with an acceptable OS (3-year OS, 38.5%) and risk of grade ≥ 3 toxicity (incidence rate, 3.5%) in metastasis-directed therapy for L-OM. This result corresponded with those of previous reports on PT for L-OM [30–35]. The prescribed dose range in this cohort series was relatively wide because the prescribed doses to treat L-OM depended on the physicians’ decisions among different facilities. However, many lesions (89.8%, 239/266) in this cohort series received PT with high prescribed doses of BED_10_ > 96 Gy (RBE). That factor seemed to be the reason the statistical analysis revealed no significance between PT series with prescribed dose < the median value or ≥ the median value. With regard to adverse events, the incidence rate of grade 3 adverse events was 3.5% in the PT series, and the dominant toxicity was dermatitis (3/7, 42.9%). No cases of grade ≥ 4 fatal adverse events occurred. The incidence of grade 3 adverse events in PT for L-OM was low and likely similar to the incidence in published X-SBRT data (0–9.8%).

The statistical comparison between all data sets and colorectal cancer data sets consistently showed that PT offered superior LC than did X-SBRT. In the PT for L-OM series, the median delivered BED_10_ was 109.6 Gy (RBE) and approximately 90% of lesions received more than 96 Gy (RBE). Previous investigations have reported that a higher delivery dose to targets is associated with better LC [22, 24]; therefore, the substantially high dose in the PT series seemed to result in better LC. For OS, the statistical comparison between all data sets of PT and X-SBRT revealed no statistically significant difference. However, the statistical comparison between the colorectal cancer data sets demonstrated that PT offered superior OS. As Klement *et al.* [29] suggested, metastatic disease control may potentially improve OS in patients with colorectal cancer. Therefore, the improved LC may have resulted in better OS in the cohort series of PT for L-OM. However, in general, the LC does not always have a direct impact on OS because OS is associated with many factors (e.g. patient and disease characteristics; combined treatment modality; treatment response, especially in systemic treatment; and follow-up time). Therefore, more precise validation with a larger sample size and appropriate adjustment for variations will be essential in future studies to evaluate the impact of PT for L-OM on survival benefit more appropriately.

This cohort data set of PT for L-OM included 38 (15.2%) lesions measuring > 5 cm and 30 (12%) lesions measuring > 6 cm among 250 measurable lesions. The analysis revealed that PT for large L-OM (i.e. tumor size > 5 cm) provided durable LC (3-year LC, 84.5%) with acceptable toxicity (i.e. incidence of grade ≥ 3 adverse events, 2.9%). In general, a large liver tumor measuring > 5 cm or 6 cm does not seem to be a good candidate for X-SBRT in the context of the risk of severe toxicity [19–21, 25, 26]. Thus, few studies have analyzed X-SBRT for large L-OMs. By contrast, our cohort data demonstrated that PT may be a safe treatment for L-OM tumors measuring > 5 cm, as well as for L-OM tumors measuring ≤ 5 cm, and it suggested that PT could be a promising treatment for large L-OM tumors measuring > 5 cm, which may be ineligible for X-SBRT.

### Lymph node oligometastasis

The analysis of the national cohort data set showed that PT offered durable LC (3-year LC, 72.8%) with acceptable OS (3-year OS, 50.2%) and incidence of grade ≥ 3 adverse effects (3.2%) in metastasis-directed therapy for LN-OM. This result corresponded with previous findings on PT for LN-OM [53–57]. In the cohort series of PT for LN-OM, the median delivered BED_10_ was 79.2 Gy (RBE). Compared to the delivery doses in the series of P-OM or L-OM, the doses of LN-OM were lower, although the range of the doses was narrower. This finding may be caused by the target location in the treatment for LN-OM. In general, in the treatment for LN-OM, the target sites are often very close to bowels, which are vulnerable to radiation. In the cohort series, 44.4% (4/9) of grade 3 late toxicities was bowel toxicity.

However, the 3-year LC rate of 72.8% in the PT series for LN-OM was comparable with the reported rates of the X-SBRT series, as the statistical comparisons of all combinations demonstrated. Franzese *et al.* [42] in their large series (278 patients with 418 lesions) reported that a delivery dose of BED_10_ ≥ 75 Gy was a significant independent factor associated with better LC. Many target lesions in the cohort LN-OM series (72.8%, 209/287) were treated with a dose of BED_10_ ≥ 75 Gy (RBE). The relatively high-dose delivery in the cohort series of PT for LN-OM probably resulted in the durable LC. The incidence of grade ≥ 3 adverse events was low in this cohort series (3.2%), as well as in previous reports on PT for LN-OM (range, 0.0–0.3%). Therefore, we concluded that PT could provide comparable LC with acceptable toxicity as that of X-SBRT or X-IMRT.

For OS, statistical analyses had inconsistent results among the different comparative combinations. In general, OS is influenced by many factors. The population with LN-OM likely has highly heterogeneous patient and lesion characteristics and treatment history. In this study, the reports included in the comparative analysis had high heterogeneity. The correction of heterogeneity consequently was not conducted because of the difficulty in appropriate adjustment, which was likely to cause inconsistent results in our analysis. More precise validation with larger sample size and appropriate adjustment for differences will be necessary in future studies. Our analysis had some limitations, although the results demonstrated that, at any rate, PT for LN-OM provided comparable OS outcomes as those of X-SBRT/X-IMRT.

This study has several limitations. The multi-institutional cohort intrinsically includes variations between the participating institutions, which may have unintentionally resulted in a significant bias. Statistical comparisons were conducted using only the data sets extracted from reports describing the events of interest. The data sets used in the statistical comparisons may be too heterogeneous to achieve statistical results with modest bias. However, metastasis-directed therapy for oligometastatic disease intrinsically has large heterogeneity. Many factors (e.g. characteristics of the patients and disease, intensity of the metastasis-directed therapy or combined therapy, treatment history before metastasis-directed therapy) can cause heterogeneity and difficulty in making an appropriate comparison with retrospective data sets. Therefore, more precise validation with prospective comparative studies is needed.

In conclusion, PT provided durable LC with acceptable OS and risk of severe toxicity in radical metastasis-directed therapy for P-OM, L-OM and LN-OM. For LC, PT, compared to X-SBRT or X-IMRT, was potentially superior for treating P-OM; was superior for treating L-OM; and was equivalent for treating LN-OM. In particular, this study showed PT may be a new treatment option for L-OM tumors measuring > 5 cm.

## Supplementary Material

Supplementary_data_1_20221030_rrad004

Supplementary_data_2_20221204_rrad004

Supplementary_data_3_20221218_rrad004
