# Supplementary material for: Comprehensive analysis of Japanese nationwide cohort data of particle beam therapy for pulmonary, liver and lymph node oligometastases: particle beam therapy versus high-precision X-ray radiotherapy
Source: J Radiat Res. 2023 Apr 13;64(Suppl 1):i69–83. doi: 10.1093/jrr/rrad004 (PMC10278882; doi:10.1093/jrr/rrad004)
Supplement: Supplementary_data_1_20221030_rrad004 [file supplementary_data_1_20221030_rrad004.docx]

**Supplemental data 1. Detailed description of the method for systematic review**

**‧ Pulmonary oligometastases**

In the systematic review of X-SBRT (X-ray stereotactic body radiotherapy) and PT (particle beam therapy), which included PBT (proton beam therapy) and C-ion RT (carbon-ion radiotherapy), for pulmonary oligometastasis (P-OM), medical literature published in English between January 2000 and September 2020 was searched in PubMed using the terms listed in Table S1a. The inclusion criteria for the literature search were defined using the population, intervention, control, outcome, study design (PICOS) approach, as shown in Table S1b. Given that most reports focusing on the outcomes of P-OM had a retrospective design and short follow-up duration with heterogeneity of primary disease sites, we defined the inclusion criteria as follows: clinical trials, prospective, and retrospective studies were eligible for analysis if they reported outcomes of local control, overall survival, or severe toxicity of grade ≥ 3 in patients receiving X-SBRT or PT with radical intent for P-OM. Two radiation oncologists independently reviewed the retrieved articles and selected potentially relevant ones based on their titles and abstracts. Finally, full-text reviews were performed to identify studies that met the selection criteria (Table S1b). The PRISMA flow diagram of the systematic review of P-OM is shown in Figure S1. The relevant data was extracted from the identified studies that met the full selection criteria. The extracted variables included study-level or patient-level characteristics such as study design, phase of trial, sample size, duration of follow-up, delivery method of radiotherapy, dose per fraction, fraction number, site of primary cancer, and site of treatment. Information regarding the endpoint outcomes of local control, overall survival, or treatment-induced toxicities of grade ≥ 3 was also extracted.

| **Table S1a. Research terms for systematic review for P-OM** |
| --- |
| - **Research terms for X-SBRT**   [“stereotactic AND radiotherapy OR radiosurgery NOT cerebral NOT brain” “lung OR pulmonary OR thoracic” “metastases OR metastatic OR oligometastases” “carbon-ion radiotherapy” “proton beam radiotherapy”] |
| - **Research terms for PT**   [“stereotactic AND radiotherapy OR radiosurgery NOT cerebral NOT brain” “lung OR pulmonary OR thoracic” “metastases OR metastatic OR oligometastases” “carbon-ion radiotherapy” “proton beam radiotherapy”] |

Abbreviations: P-OM, pulmonary oligometastasis; X-SBRT, x-ray stereotactic body radiotherapy; PT, particle beam therapys

| **Table S1b. Population, Intervention, Control, Outcome, Study Design (PICOS) inclusion criteria** | |
| --- | --- |
| **Population** | Patients diagnosed with P-OM. |
| **Intervention** | X-SBRT or PT with radical intent. |
| **Control** | Control group was not stipulated. |
| **Outcomes** | The study must report at least one of the following outcomes: local control rate of P-OM, overall survival rate, median survival time, incidence of any toxicity of grade ≥ 3. |
| **Study design** | For X-SBRT, clinical trials, prospective, and retrospective studies were eligible, although a study must include at least 40 patients in the screening review and at least 300 patients in the full-text review. As an exception, 60-patient articles focusing on primary cancer of lung were eligible because the statistical comparison was made between the datasets of the same primary cancer of the X-SBRT and the PT to minimize the impact of primary cancer on the values of interest (local progression rate or mortality).  For PT, clinical trials, prospective, and retrospective studies were eligible, while case reports were excluded. |

Abbreviations: P-OM, pulmonary oligometastasis; X-SBRT, x-ray stereotactic body radiotherapy; PT, particle beam therapy

**Figure S1. Inclusion flow diagram for the selection of articles included in the systematic review of the radical-intent radiotherapy for P-OM.**


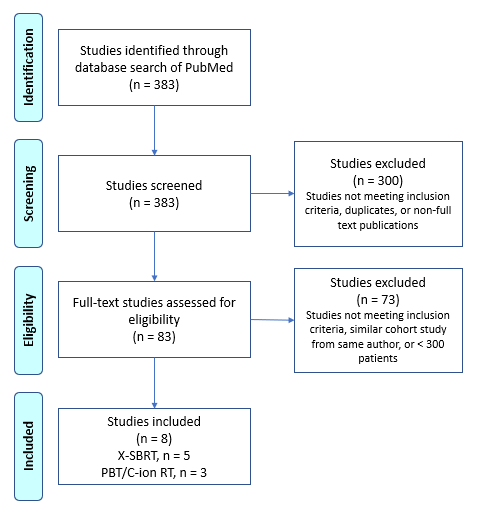


Abbreviations: P-OM, pulmonary oligometastasis; X-SBRT, x-ray stereotactic body radiotherapy; PBT, proton beam therapy; C-ion RT, carbon-ion radiotherapy

**‧ Liver oligometastasis**

In the systematic review of X-SBRT and PT (PBT/C-ion RT) for liver oligometastasis (L-OM), medical literature published in English between January 2000 and September 2020 was searched in PubMed using the terms listed in Table S2a. The inclusion criteria for the literature search were defined using the PICOS approach (Table S2b). Given that most reports focusing on the outcomes of L-OM had a retrospective design and short follow-up duration with heterogeneity of primary disease sites, we defined the inclusion criteria as follows: for X-SBRT, prospective phase II studies, and retrospective multi-institutional studies were eligible for analysis if they reported outcomes of local control, overall survival, or severe toxicity of grade ≥ 3 in patients receiving X-SBRT with radical intent for L-OM. For PT, clinical trials, prospective, and retrospective studies were eligible for analysis if they reported outcomes of local control, overall survival, or severe toxicity of grade ≥ 3 in patients receiving PT with radical intent for L-OM. Two radiation oncologists independently reviewed the retrieved articles and selected potentially relevant ones based on their titles and abstracts. Finally, full-text reviews were performed to identify studies that met the selection criteria. The PRISMA flow diagram of the systematic review of the L-OM is illustrated in Figure S2. The relevant data was extracted from the identified studies that met the full selection criteria. The extracted variables included study-level or patient-level characteristics such as study design, phase of trial, sample size, duration of follow-up, delivery method of radiotherapy, dose per fraction, fraction number, site of primary cancer, and site of treatment. Information regarding the endpoint outcomes of local control, overall survival, and treatment-induced toxicities of grade ≥ 3 was also extracted.

| **Table S2a. Research terms for systematic review for L-OM** |
| --- |
| - **Research terms for X-SBRT**   (“stereotactic AND radiotherapy OR radiation therapy OR  SRT OR　SBRT OR　SABR OR radiosurgery, NOT cerebral NOT brain” “liver OR hepatic” “metastases OR metastatic OR oligometastases”) |
| - **Research terms for PT**   (“liver OR hepatic” [Title/Abstract] OR “adenopath*”[Title/Abstract]) AND (((((“cancer”[Title/Abstract] AND “cancers”[Title/Abstract]) OR “neoplasm”[Title/Abstract]) AND “neoplasms”[Title/Abstract]) OR “neoplasms”[MeSH Terms] OR “tumor”[Title/Abstract] OR “tumors”[Title/Abstract] OR “tumour”[Title/Abstract] OR “carcinoma”[Title/Abstract] OR “carcinomas”[Title/Abstract]) AND (“English”[Language] AND 2000/01/01:2021/12/31[Date - Publication])) AND ((“metastasis”[Title/Abstract] OR “metastases”[Title/Abstract] OR “Neoplasm Metastasis”[MeSH Terms] OR “oligometastasis”[Title/Abstract] OR “oligometastases”[Title/Abstract]) AND “English”[Language]) AND ((“proton therapy”[Title/Abstract] OR “proton radiotherapy”[Title/Abstract] OR “proton beam therapy”[Title/Abstract] OR “proton beam radiotherapy”[Title/Abstract] OR “carbon ion therapy”[Title/Abstract] OR “carbon ion radiotherapy”[Title/Abstract] OR “carbon ion beam therapy”[Title/Abstract] OR “carbon ion beam radiotherapy”[Title/Abstract] OR “heavy ion radiotherapy”[Title/Abstract] OR “heavy ion radiotherapy”[MeSH Terms] OR “hadron therapy”[Title/Abstract] OR “hadrontherapy”[Title/Abstract]) AND “english”[Language]). |

Abbreviations: L-OM, liver oligometastasis; X-SBRT, x-ray stereotactic body radiotherapy; PT, particle beam therapy

| **Table 2b. Population, Intervention, Control, Outcome, Study Design (PICOS) inclusion criteria** | |
| --- | --- |
| **Population** | Patients diagnosed with L-OM. |
| **Intervention** | X-SBRT or PT with radical intent. |
| **Control** | Control group was not stipulated. |
| **Outcomes** | The study must report at least one of the following outcomes: local control rate of L-OM, overall survival rate, median survival time, incidence of any toxicity of grade ≥ 3. |
| **Study design** | For X-SBRT, prospective phase II studies, and retrospective multi-institutional studies were eligible for the full-text review.  For PT, clinical trials, prospective, and retrospective studies were eligible, while case reports were excluded. |

Abbreviations: L-OM, liver oligometastasis; X-SBRT, x-ray stereotactic body radiotherapy; PT, particle beam therapy

**Figure S2. Inclusion flow diagram for the selection of articles included in the systematic review of the radical-intent radiotherapy for L-OM.**


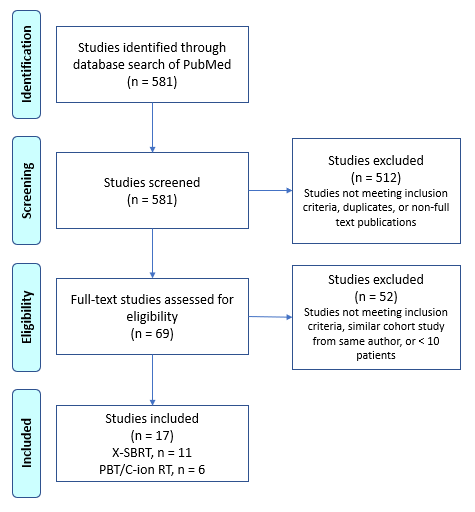


Abbreviations: L-OM, liver oligometastasis; X-SBRT, x-ray stereotactic body radiotherapy; PBT, proton beam therapy; C-ion RT, carbon-ion radiotherapy

**‧ Lymph node oligometastasis**

In the systematic review of X-SBRT/X-IMRT and PT (PBT/C-ion RT) for lymph node oligometastasis (LN-OM), medical literature published in English between January 2000 and September 2020 was searched in PubMed using the terms listed in Table S3a. The inclusion criteria for the literature search were defined using the PICOS approach (Table S3b). Given that most reports focusing on the outcomes of LN-OM had a retrospective design and short follow-up duration with a heterogeneity in primary disease sites, we defined the inclusion criteria as follows: clinical trials, prospective studies, and retrospective studies were eligible for analysis if they reported outcomes of local control, overall survival, or severe toxicity of grade ≥ 3 in patients receiving X-SBRT, X-IMRT, or PT with radical intent for LN-OM. Two radiation oncologists independently reviewed the retrieved articles and selected potentially relevant ones based on their titles and abstracts. Finally, full-text reviews were performed to identify studies that met the selection criteria (Table S3b). The PRISMA flow diagram of the systematic review for LN-OM is illustrated in Figure S3. The relevant data was extracted from the identified studies that met the full selection criteria. The extracted variables included study-level or patient-level characteristics such as study design, phase of trial, sample size, duration of follow-up, delivery method of radiotherapy, dose per fraction, fraction number, site of primary cancer, and site of treatment. Information regarding the endpoint outcomes of local control, overall survival, and treatment-induced toxicities of grade ≥ 3 was also extracted.

| **Table S3a. Research terms for systematic review for LN-OM** |
| --- |
| - **Research terms for X-SBRT/X-IMRT**   (“lymph”[Title/Abstract] OR “adenopath*”[Title/Abstract]) AND (((((“cancer”[Title/Abstract] AND “cancers”[Title/Abstract]) OR “neoplasm”[Title/Abstract]) AND “neoplasms”[Title/Abstract]) OR “neoplasms”[MeSH Terms] OR “tumor”[Title/Abstract] OR “tumors”[Title/Abstract] OR “tumour”[Title/Abstract] OR “carcinoma”[Title/Abstract] OR “carcinomas”[Title/Abstract]) AND (“english”[Language] AND 2000/01/01:2021/12/31[Date - Publication])) AND ((“metastasis”[Title/Abstract] OR “metastases”[Title/Abstract] OR “Neoplasm Metastasis”[MeSH Terms] OR “oligometastasis”[Title/Abstract] OR “oligometastases”[Title/Abstract]) AND “english”[Language]) AND (“SRT”[Title/Abstract] OR “SBRT”[Title/Abstract] OR “SABR”[Title/Abstract] OR “IMRT”[Title/Abstract] OR “radiosurgery”[Title/Abstract] OR “radiosurgery”[MeSH Terms]) |
| - **Research terms for PT**   (“lymph”[Title/Abstract] OR “adenopath*”[Title/Abstract]) AND (((((“cancer”[Title/Abstract] AND “cancers”[Title/Abstract]) OR “neoplasm”[Title/Abstract]) AND “neoplasms”[Title/Abstract]) OR “neoplasms”[MeSH Terms] OR “tumor”[Title/Abstract] OR “tumors”[Title/Abstract] OR “tumour”[Title/Abstract] OR “carcinoma”[Title/Abstract] OR “carcinomas”[Title/Abstract]) AND (“english”[Language] AND 2000/01/01:2021/12/31[Date - Publication])) AND ((“metastasis”[Title/Abstract] OR “metastases”[Title/Abstract] OR “Neoplasm Metastasis”[MeSH Terms] OR “oligometastasis”[Title/Abstract] OR “oligometastases”[Title/Abstract]) AND “english”[Language]) AND ((“proton therapy”[Title/Abstract] OR “proton radiotherapy”[Title/Abstract] OR “proton beam therapy”[Title/Abstract] OR “proton beam radiotherapy”[Title/Abstract] OR “carbon ion therapy”[Title/Abstract] OR “carbon ion radiotherapy”[Title/Abstract] OR “carbon ion beam therapy”[Title/Abstract] OR “carbon ion beam radiotherapy”[Title/Abstract] OR “heavy ion radiotherapy”[Title/Abstract] OR “heavy ion radiotherapy”[MeSH Terms] OR “hadron therapy”[Title/Abstract] OR “hadrontherapy”[Title/Abstract]) AND “english”[Language]). |

Abbreviations: LN-M, lymph node oligometastasis; X-SBRT, x-ray stereotactic body radiotherapy; X-IMRT, x-ray intensity-modulated radiotherapy; PT, particle beam therapy

| **Table S3b. Population, Intervention, Control, Outcome, Study Design (PICOS) inclusion criteria** | |
| --- | --- |
| **Population** | Patients diagnosed with LN-OM. |
| **Intervention** | X-SBRT, X-IMRT, or PT with radical intent. |
| **Control** | Control group was not stipulated. |
| **Outcomes** | The study must report at least one of the following outcomes: local control rate of LN-OM, overall survival rate, median survival time, incidence of any toxicity of grade ≥ 3. |
| **Study design** | For X-SBRT/X-IMRT or PT, clinical trials, prospective, and retrospective studies were eligible, although a study must include at least 5 patients in the screening review and at least 10 patients in the full-text review. |

Abbreviations: LN-OM, lymph node oligometastasis; X-SBRT, x-ray stereotactic body radiotherapy; X-IMRT, x-ray intensity-modulated radiotherapy; PT, particle beam therapy

**Figure S3. Inclusion flow diagram for the selection of articles included in the systematic review of the radical-intent radiotherapy for LN-OM.**

**
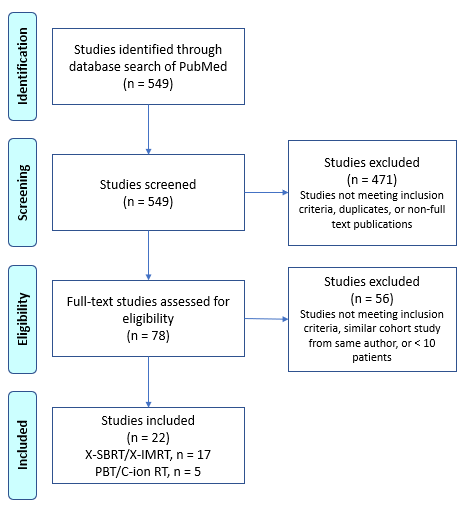
**

Abbreviations: LN-OM, lymph node oligometastasis; X-SBRT, x-ray stereotactic body radiotherapy; X-IMRT, x-ray intensity-modulated radiotherapy; PBT, proton beam therapy; C-ion RT, carbon-ion radiotherapy
