# Supplementary material for: Comprehensive analysis of Japanese nationwide cohort data of particle beam therapy for pulmonary, liver and lymph node oligometastases: particle beam therapy versus high-precision X-ray radiotherapy
Source: J Radiat Res. 2023 Apr 13;64(Suppl 1):i69–83. doi: 10.1093/jrr/rrad004 (PMC10278882; doi:10.1093/jrr/rrad004)
Supplement: Supplementary_data_2_20221204_rrad004 [file supplementary_data_2_20221204_rrad004.docx]

**Supplementary data 2.**

**The dose fractionation schemes of particle therapy for pulmonary, liver, and lymph node oligometastases**

| Dose fractionation schemes, n (%) | | | | |
| --- | --- | --- | --- | --- |
| P-OM  132 patients (156 tumors) |  | **L-OM**  **200 patients (266 tumors)** |  | **LN-OM**  **282 patients (287 regions)** |
| C-ion RT (49 tumors)  50.0 Gy(RBE)/ 1fr. 4 (8.2)  54.0 Gy(RBE)/ 4fr. 1 (2.0)  60.0 Gy(RBE)/ 4fr. 30 (61.2)  68.4 Gy(RBE)/ 12fr. 2 (4.1)  69.6 Gy(RBE)/ 12fr. 3 (6.1)  72.0 Gy(RBE)/ 12fr. 6 (12.2)  64.0 Gy(RBE)/ 16fr. 1 (2.0)  70.4 Gy(RBE)/ 16fr. 1 (2.0)  72.0 Gy(RBE)/ 16fr. 1 (2.0) |  | **C-ion RT (58 tumors)**  58.0 Gy(RBE)/ 1fr. 4 (6.9)  60.0 Gy(RBE)/ 4fr. 42 (72.4)  64.0 Gy(RBE)/ 8fr. 3 (5.2)  68.0 Gy(RBE)/ 8fr. 1 (1.7)  60.0 Gy(RBE)/ 12fr. 1 (1.7)  64.8 Gy(RBE)/ 12fr. 1 (1.7)  68.0 Gy(RBE)/ 16fr. 3 (5.2)  76.0 Gy(RBE)/ 20fr. 3 (5.2) |  | **C-ion RT (82 regions)**  48.0 Gy(BRE)/ 12fr. 7 (8.5)  51.6 Gy(RBE)/ 12fr. 1 (1.2)  52.8 Gy(RBE)/ 12fr. 38 (46.3)  55.2 Gy(RBE)/ 12fr. 18 (22.0)  57.6 Gy(RBE)/ 16fr. 18 (22.0) |
| PBT (107 tumors)  64.0 Gy(RBE)/ 8fr. 56 (52.3)  66.0 Gy(RBE)/ 10fr. 25 (23.4)  70.0 Gy(RBE)/ 10fr. 1 (0.9)  80.0 Gy(RBE)/ 10fr. 1 (0.9)  72.6 Gy(RBE)/ 22fr. 20 (18.7)  80.0 Gy(RBE)/ 25fr. 1 (0.9)  70.0 Gy(RBE)/ 35fr. 3 (2.8) |  | **PBT (208 tumors)**  64.0 Gy(BRE)/ 8fr. 64 (30.8)  66.0 Gy(RBE)/ 10fr. 46 (22.1)  72.6 Gy(RBE)/ 22fr. 69 (33.2)  74.0 Gy(RBE9/ 37fr. 29 (13.9) |  | **PBT (205 regions)**  66.0 Gy(RBE)/ 10fr. 1 (0.5)  59.4 Gy(RBE)/ 18fr. 1 (0.5)  66.0 Gy(RBE)/ 20fr. 1 (0.5)  72.6 Gy(RBE)/ 22fr. 31 (15.1)  72.0 Gy(RBE)/ 24fr. 1 (0.5)  50.0 Gy(RBE)/ 25fr. 25 (12.2)  62.5 Gy(RBE)/ 25fr. 1 (0.5)  52.0 Gy(RBE)/ 26fr. 1 (0.5)  65.0 Gy(RBE)/ 25fr. 26 (12.7)  54.0 Gy(RBE)/ 27fr. 1 (0.5)  56.0 Gy(RBE)/ 28fr. 6 (2.9)  60.0 Gy(RBE)/ 30fr. 34 (16.6)  66.0 Gy(RBE)/ 30fr. 1 (0.5)  62.0 Gy(RBE)/ 31fr. 2 (1.0)  64.0 Gy(RBE)/ 32fr. 2 (1.0)  66.0 Gy(RBE)/ 33fr. 21 (10.2)  68.0 Gy(BRE)/ 34fr. 1 (0.5)  70.0 Gy(RBE)/ 35fr. 48 (23.4)  74.0 Gy(RBE)/ 37fr. 1 (0.5) |

Abbreviations: P-OM, pulmonary oligometastasis; L-OM, liver oligometastasis; LN-OM, lymph node oligometastasis; PBT, proton beam therapy; C-ion RT, carbon-ion radiotherapy; RBE, relative biological effectiveness; X Gy(RBE)/ Y fr., X Gy(RBE) in Y fractions
