# Supplementary material for: Comprehensive analysis of Japanese nationwide cohort data of particle beam therapy for pulmonary, liver and lymph node oligometastases: particle beam therapy versus high-precision X-ray radiotherapy
Source: J Radiat Res. 2023 Apr 13;64(Suppl 1):i69–83. doi: 10.1093/jrr/rrad004 (PMC10278882; doi:10.1093/jrr/rrad004)
Supplement: Supplementary_data_3_20221218_rrad004 [file supplementary_data_3_20221218_rrad004.docx]

**Supplementary data 3.**

**Results of comparison between the same specific primary cancer data sets of the cohort data of PT and those of the extracted historical data of X-SBRT/X-IMRT, based on the systematic review**

|  | **Data sets for comparison** | **Treatment modality**  **(Number of patients / target sites)** | **Local progression** | | **Mortality** | | **Incidence of**  **G≥3 AEs (%)** |
| --- | --- | --- | --- | --- | --- | --- | --- |
|  |  |  | **IRR (95% CI)** | ***P* value** | **IRR (95% CI)** | ***P* value** |  |
| **P-OM** | Colorectal cancer | PT (44 / 47) | 0.83 (0.46-1.51) | 0.542 | 0.59 (0.31-1.13) | 0.110 | 0 |
|  |  | X-SBRT (330/ 371) |  |  |  |  | 1.5-2.2 |
|  | Lung cancer | PT (35 / 35) | 0.92 (0.24-3.56) | 0.904 | 0.48 (0.23-1.02) | 0.056 | 0 |
|  |  | X-SBRT (60 / 90) |  |  |  |  | 3.3 |
| **L-OM** | Colorectal cancer | PT (102/ 130) | 0.33 (0.22-0.50) | < 0.001 | 0.69 (0.49-0.97) | 0.034 | 2.9 |
|  |  | X-SBRT (165/ 236) |  |  |  |  | 0 |
| **LN-OM** | Colorectal cancer | PT (43/ 43) | 0.88 (0.33-2.34) | 0.798 | 2.39 (0.74-7.77) | 0.148 | 2.3 |
|  |  | X-SBRT/X-IMRT (35/ 38) |  |  |  |  | 0 |
|  | Lung cancer | PT (37/ 39) | 0.54 (0.16-1.80) | 0.316 | 0.45 (0.26-0.79) | 0.006 | 5.4 |
|  |  | X-SBRT/X-IMRT (60/ 63) |  |  |  |  | 3.7-6.1 |
|  | Uterine cancer | PT (38/ 39) | 0.49 (0.19-1.24) | 0.134 | 0.66 (0.39-1.13) | 0.126 | 7.9 |
|  |  | X-SBRT/X-IMRT (153/ 168) |  |  |  |  | 3.3-21.1 |

Abbreviations: PT, particle beam therapy; P-OM; pulmonary oligometastasis; L-OM, liver oligometastasis; LN-OM, lymph node oligometastasis; PBT, proton beam therapy; C-ion RT, carbon-ion radiotherapy; X-SBRT, x-ray stereotactic body radiotherapy; G≥3 AEs, adverse effects of grade≥3; IRR, incidence rate ratio; CI, confidence interval.
